# SLIT3 promotes cardiac fibrosis and differentiation of cardiac fibroblasts by RhoA/ROCK1 signaling pathway

**DOI:** 10.22038/IJBMS.2024.73812.16044

**Published:** 2024

**Authors:** Xiaogang Zhang, Bei Tian, Xinpeng Cong, Zhongping Ning

**Affiliations:** 1 Department of Cardiology, Shanghai Pudong New Area Zhoupu Hospital (Zhoupu Hospital affiliated to Shanghai Medical College of Health), Pudong New District, Shanghai 201318, China; # These authors contributed equally to this work

**Keywords:** α-Smooth muscle actin, Cardiac fibroblast, Cardiac fibrosis, Collagen I, Collagen III, Slit guidance ligand 3

## Abstract

**Objective(s):**

Slit guidance ligand 3 (SLIT3) has been identified as a potential therapeutic regulator against fibroblast activity and fibrillary collagen production in an autocrine manner. However, this research aims to investigate the potential role of SLIT3 in cardiac fibrosis and fibroblast differentiation and its underlying mechanism.

**Materials and Methods:**

C57BL/6 mice (male, 8-10 weeks, n=47) were subcutaneously infused with Ang II (2.0 mg/kg/day) for 4 weeks. One to two-day-old Sprague-Dawley (SD) rats were anesthetized by intraperitoneal injection of 1% pentobarbital sodium (60 mg/kg) and ketamine (50 mg/kg) and the cardiac fibroblast was isolated aseptically. The mRNA and protein expression were analyzed using RT-qPCR and Western blotting.

**Results:**

The SLIT3 expression level was increased in Ang II-induced mice models and cardiac fibroblasts. SLIT3 significantly increased migrated cells and α-smooth muscle actin (α-SMA) expression in cardiac fibroblasts. Ang II-induced increases in mRNA expression of collagen I (COL1A1), and collagen III (COL3A1) was attenuated by SLIT3 inhibition. SLIT3 knockdown attenuated the Ang II-induced increase in mRNA expression of ACTA2 (α-SMA), Fibronectin, and CTGF. SLIT3 suppression potentially reduced DHE expression and decreased malondialdehyde (MDA) content, and the superoxide dismutase (SOD) and catalase (CAT) levels were significantly increased in cardiac fibroblasts. Additionally, SLIT3 inhibition markedly decreased RhoA and ROCK1 protein expression, whereas ROCK inhibitor Y-27632 (10 μM) markedly attenuated the migration of cardiac fibroblasts stimulated by Ang II and SLIT3.

**Conclusion:**

The results speculate that SLIT3 could significantly regulate cardiac fibrosis and fibroblast differentiation via the RhoA/ROCK1 signaling pathway.

## Introduction

Cardiac fibrosis is crucial to cardiac remodeling after hypertension, myocardial infarction, and other cardiovascular disorders (1). An essential step in the development of cardiac fibrosis is the differentiation of cardiac fibroblasts into myofibroblasts, which are distinguished by the expression of α-smooth muscle actin (α-SMA) and the formation of extracellular matrix (ECM) elements such as collagen types I and III (2, 3). Angiotensin II (Ang II), which is abnormally activated in several cardiovascular disorders, including hypertension and myocardial infarction, contributes significantly to cardiac fibrosis by stimulating myofibroblast development (4, 5). Therefore, reducing the Ang II-induced myofibroblast differentiation might be an essential strategy for improving cardiac fibrosis.

In cell migration and differentiation, reactive oxygen species (ROS) are recognized to be essential signaling and regulatory molecules (6). According to several recent studies, ROS are crucial in developing fibrotic disorders such as renal, hepatic, and pulmonary fibrosis (7-9). It is feasible that Ang II-induced myofibroblast differentiation is mediated via the activation of ROS generation, given that ROS are produced by Ang II stimulation in various cells.

Rho, a GTPase from the Ras superfamily with a relatively low molecular mass, is a member of the Rho subfamily (10). RhoA possesses both GDP/GTP binding and GTPase activity. This molecular switch between the GDP-inactivation state (GDP-RHO) and the GTP-activation state (GTP-RHO) cyclically controls RhoA activity (11). Guanine nucleotide exchange factors can cause GDP-RHO to transform into GTP-RHO when various receptor agonists, including Ang II and TGF-β1, activate cells. Then, GTP-RHO interacts with its particular target by attaching to the cell membrane via its C-terminal tail (11, 12). ROCK is a critical player in myocardial fibrosis and was the first downstream Rho A kinase discovered (13). There are two isomers in the ROCK family: ROCK1 and ROCK2 (12). The development of cardiac fibroblast cells can be aided by activated ROCK1, contributing to non-adaptive myocardial fibrosis (14, 15). Additionally, it can cause cells to secrete TGF-β1, CTGF, and other pro-fibrosis factors that encourage the production of type I and type III collagen in the ECM, thus fostering fibrosis (16-18). One recent study reported that ROS and the RhoA/ROCK pathway are related because inflammatory atherosclerotic lesions increase ROCK, whereas treatment of a RhoA/ROCK inhibitor decreases atherosclerotic plaque growth and apoptosis in rats (19). However, therapeutic intervention in the RhoA/ROCK1 signaling pathway could significantly manage hypertension-induced cardiac fibrosis.

Slit guidance ligand proteins known as SLITs, including SLIT1, SLIT2, and SLIT3, were discovered to be highly conserved in several species (20). In the early stages, SLITs were identified as chemorepellents that regulated axon crossing in the middle of the brain (21). However, they have been shown to significantly regulate several cellular processes in various tissue types, including the heart, mammary glands, lungs, and kidneys (22, 23). According to the results of a recent study, SLIT3 regulates organogenesis throughout embryonic development and functions as a potent angiogenic factor (24). According to an analysis of GEO data (GSE163211), SLIT3 is up-regulated in the liver tissue of people with fibrosing non-alcoholic steatohepatitis (25). A recent study reported that SLIT3 inhibits fibrillar collagen formation, controls YAP transcription, and contributes to poor remodeling and stress overload-induced cardiac fibrosis (26). However, SLIT3 could potentially affect cardiac fibrosis and the differentiation of cardiac fibroblasts. Therefore, this study aims to explore and provide precise information about the potential effect of SLIT3 regarding cardiac fibrosis and the differentiation of cardiac fibroblasts and to investigate the specific molecular mechanism.

## Materials and Methods

***Animals model of cardiac fibrosis***

C57BL/6 mice (male, 8-10 weeks, n=47) were used to establish the cardiac fibrosis model. They were subcutaneously infused with Ang II (2.0 mg/kg/day) or the same volume saline using osmotic mini-pumps for 4 weeks. The systolic blood pressure (SBP) was measured by a tail-cuff system (Softron BP98A; Softron Tokyo, Japan) on day 28. This study received approval from the Animal Care and Use Committee of Shanghai Pudong Zhoupu Hospital and adhered to the guidelines outlined in the Guide for the Care and Use of Laboratory Animals.

***Sample collection***

The ventricular tissue was collected after mice were anesthetized with 1% pentobarbital sodium (50 mg/kg). Some tissue was snap-frozen in liquid nitrogen for RT-qPCR and western blot analysis, and another tissue was fixed in 4% paraformaldehyde for histological studies. Masson’s trichrome staining validated cardiac fibrosis.

***Cardiac fibrosis assessment***

The cardiac fibrosis was evaluated by Masson trichrome staining. The fibrosis extent was evaluated by the ratio of the fibrotic area to normal myocardium [fibrotic area (%)].

***Isolation of rat cardiac fibroblasts***

The primary rat cardiac fibroblasts were isolated according to the published protocol (27). The 1 to 2-day newborn Sprague-Dawley (SD) rats were anesthetized by intraperitoneal injection of 1% pentobarbital sodium (60 mg/kg) and ketamine (50 mg/kg) before thoracotomy under sterile conditions. Then, the heart was isolated aseptically and placed in a petri dish. The ventricular tissues were isolated from the heart and washed to remove congestion and excess epicardial tissues. After that, ventricular tissues were cut into pieces of about 1 mm^3^ in size, followed by trypsin digestion and differential centrifugation. The cardiac fibroblasts were obtained and infused in DMEM (Gibco, Grand Island, NY, USA) with 10% fetal bovine serum for incubation at 37 ^°^C with 5% CO_2_. The culture medium was replaced every 3 days, the cell morphology was observed, and pictures were captured under an inverted microscope.

***Cell culture***

Cells were cultured in the DMEM containing 10% (vol/vol %)-fetal bovine serum (FBS) in a humidified atmosphere at 37 ^°^C with 5% CO_2_. Cells in passages 2-3 were used in experiments. Cells were treated with or without Ang II (1 μM) for 48 hr on reaching 70% confluence. Y-27632 (10 μM, HY-10071, MedChemExpress) was used to inhibit ROCK activity for the inhibitor experiment.

***RNA interference***

Small interfering RNAs (siRNAs) used to knock down rat SLIT3 were purchased from Saisuofei Biotechnology Co., Ltd (Jiangsu, China). According to the manufacturer’s instructions, GMCs were transfected with SLIT3 siRNA and NC siRNA using Vigofect (Vigorous Biotechnology, China) transfection reagent at a final concentration of 50 nM. The sequences of the siRNAs were as follows: SLIT3-1229, forward: 5′-GCC UAG AAC AGA ACU CCA UTT-3′, reverse: 5′-AUG GAG UUC UGU UCU AGG CTT-3′; SLIT3-2108, forward: 5′-GCC UGA AGA CAC UGA UGU UTT-3′, reverse: 5′-AAC AUC AGU GUC UUC AGG CTT-3′; SLIT3-3331, forward: 5′-GGG AUC AAC AAC UAC GCA UTT-3′, reverse: 5′-AUG CGU AGU UGU UGA UCC CTT-3′. Transfection was performed with Lipofectamine 2000 (Invitrogen, USA) according to the manufacturer’s instructions.

***Cell migration assay***

The migration capability of cardiac fibroblast cells was assessed by Transwell assay. Cells were placed into the top chamber of Transwell chambers (8 μm, 1×10^5^ cells/ml) in 100-μl DMEM medium without FBS. The lower chamber contained 600 μl DMEM medium with 10% FBS. At 72 hr of incubation, cells in the upper chamber were wiped with a cotton swab. Crystal violet 0.1% was used to stain the migrating cells. The stained migrated cells were counted under a light microscope (magnification, ×200) from 5 fields to the calculated average number of migrated cells.

***Immunohistochemistry***

Cells were first fixed with 4% paraformaldehyde and then made permeable with 0.1% Triton X-100 in PBS before being blocked with 3% BSA in PBS for 30 min. Primary antibodies against SLIT3 (ab307436, Abcam, UK) were then used to stain the cells at a 1:400 dilution in a blocking solution at 4 ^°^C overnight. The appropriate secondary antibody, donkey anti-rabbit IgG (H+L) Highly Cross-Adsorbed Alexa Fluor Plus 488 (catalog A32790, Invitrogen, Thermo Fisher Scientific), was added to the cells, and they were rinsed before being incubated for 60 min at room temperature in the dark. 1 μg/ml DAPI was used to label the nuclei. Using a Nikon A1 confocal microscope, pictures were captured.

***Cellular immunofluorescence***

The Immunofluorescence procedure was carried out as previously explained (28). Rat cardiac fibroblasts were acquired in frozen sections. The sections were stained with TUNEL (C1086, Beyotime) or Vimentin (ab92547, Abcam) and DAPI (S0063, Beyotime) and examined under an inverted microscope (IX51, Olympus, Japan) after being washed three times in phosphate-buffered saline (PBS).

***Intracellular ROS level evaluation***

Rat cardiac fibroblasts were incubated with DCFH-DA (1:1000) at 37 ^°^C for 30 min in the dark. Then, the samples were washed three times with PBS. Intracellular ROS levels were measured at 485 nm (excitation) and 535 nm (emission) using a microplate reader (IX71, Olympus, Japan).

***Oxidative stress***

The cardiac fibroblasts were homogenized to obtain cell lysate and preserved in the refrigerator at -80 ^°^C. The activities of MDA (S0131S, Beyotime, Shanghai, China) content, SOD (S0109, Beyotime) activity, and CAT (S0051, Beyotime) activity were determined using a commercial kit, and a previously reported protocol was implemented for the oxidative stress level (29).

***Quantitative reverse transcription polymerase chain reaction (RT-qPCR)***

Total RNA was extracted from cardiac fibroblasts using TRizol (Invitrogen, USA). Total RNA was reverse-transcribed into complementary DNA (cDNA). SYBR Green reagent (TaKaRa, Japan) was used in an ABI Prism 7700 Real-Time PCR equipment (Applied Biosystems, USA) to amplify the mRNA by Real-Time Quantitative PCR (RT-qPCR). The relative gene expression was normalized to the internal control, GADPH, using the 2-ΔΔCt formula (30). The primers for Mouse SLIT3, Mouse RhoA, Rat SLIT3, Rat Collagen I, Rat Collagen III, Rat ACTA2 (α-SMA), Rat Fibronectin, Rat CTGF, and Rat GAPDH were designed by the NCBI Primer-Blast Tool (https://www.ncbi.nlm.nih.gov/tools/primer-blast/), which is listed in Table 1.

***Western blotting***

The protein was extracted with RIPA buffer, and after centrifuging the lysates to produce the supernatants, the protein concentrations could be measured using a BCA protein assay kit. The 50 μg of protein samples were loaded for 10% SDS-PAGE electrophoresis before being transferred to a PVDF membrane (Millipore, Bedford, MA, USA). A TBS solution containing 5% skim milk blocked the membrane. The membrane was incubated with the following: SLIT3 (1:400; ab11018; rabbit polyclonal, Abcam), RhoA (1:500, sc-418, mouse monoclonal, Santa Cruz), ROCK1 (1:500, sc-17794, mouse monoclonal, Santa Cruz), and GAPDH (1:1000, ab9485, rabbit polyclonal, Abcam) antibodies overnight at 4 ^°^C. Using HRP-conjugated secondary antibodies (1:2000), the immunological reactivity of these target proteins was identified. The protein band was observed using ECL (Thermo, Waltham, MA, USA), and band density was measured using image processing software (Bio-Rad, Hercules, CA, USA).

***Statistical analyses***

We analyzed the data using GraphPad Prism 5.0 (GraphPad Software Inc., San Diego, CA, United States). Continuous variables were shown as mean±SD. One-way analysis of variance (ANOVA) or the Student’s t-test was used to make statistical comparisons. At least three times, each of the tests was repeated. The cutoff for statistical significance was *P*<0.05.

## Results

***SLIT3 expression was increased in Ang II-induced mice***

**Figure 1:**
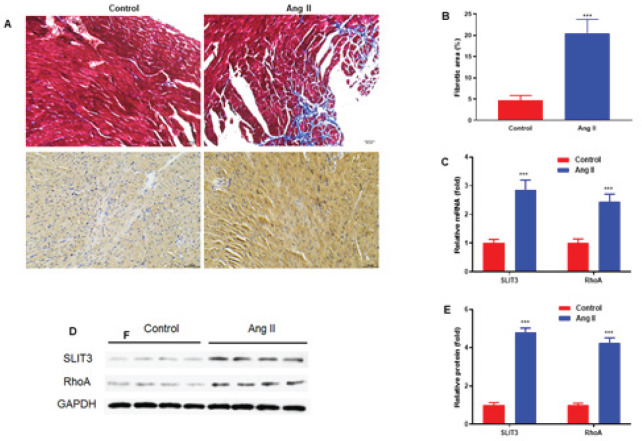
SLIT3 expression was elevated in Ang II-induced mice

To examine the expression levels of SLIT3 in Ang II-induced mice, we treated mice with Ang II (2.0 mg/kg/day) or the same volume of saline for 28 days. We examined the extent of cardiac fibrosis using Masson trichrome staining and immunocytochemistry of SLIT3 in the right ventricular myocardium (200×)(Figure 1A). Ang II significantly increased the atrial fibrotic area compared to the control mice (Figure 1B). The results also revealed that SLIT3 and RhoA mRNA levels were potentially increased in ventricular muscle from mice with Ang II infusion compared with control mice (n=4 in each group) (Figure 1C). The same trends were observed in the western blot analysis (Figures 1D and 1E). The results demonstrated that SLIT3 expressions were elevated in Ang II-induced mice.

***SLIT3 expression was increased in Ang II-induced rat cardiac fibroblasts***

**Figure 2:**
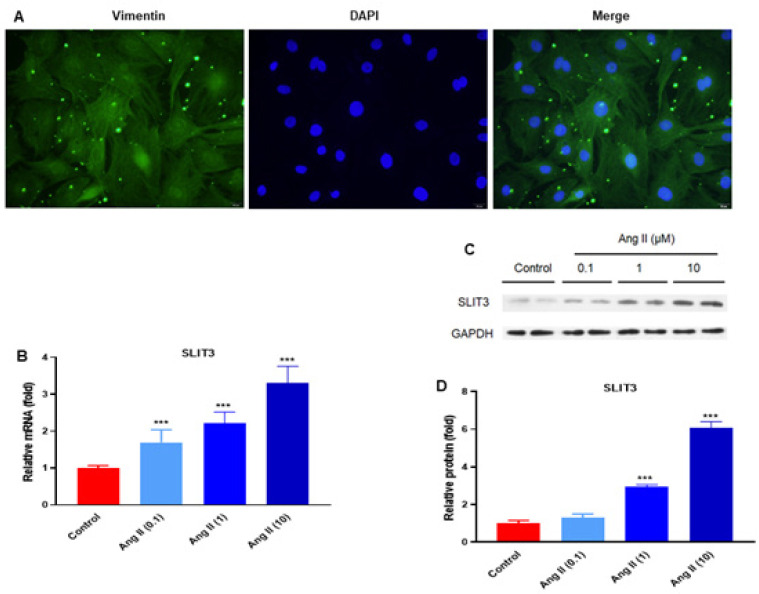
SLIT3 expression was elevated in Ang II-induced rat cardiac fibroblasts

Cardiac fibroblasts were isolated from 1 to 2-day-old newborn SD rats (lactating mice born within three days) using the differential adhesion method and were identified using Vimentin staining (400×)(Figure 2A). Then, the cardiac fibroblasts were treated with Ang II at 0.1, 1, and 10 μM for 48 hr. The results showed that SLIT3 mRNA levels were significantly increased in cardiac fibroblasts after Ang II treatment in a concentration-dependent manner (Figure 2B). SLIT3 protein expression was analyzed using western blot analysis. The results showed that SLIT3 protein levels were significantly increased in Ang II-induced cardiac fibroblasts (n=3 in each group)(Figure 2C, 2D). 

***Migration and differentiation of rat cardiac fibroblasts were accelerated by SLIT3***

**Figure 3:**
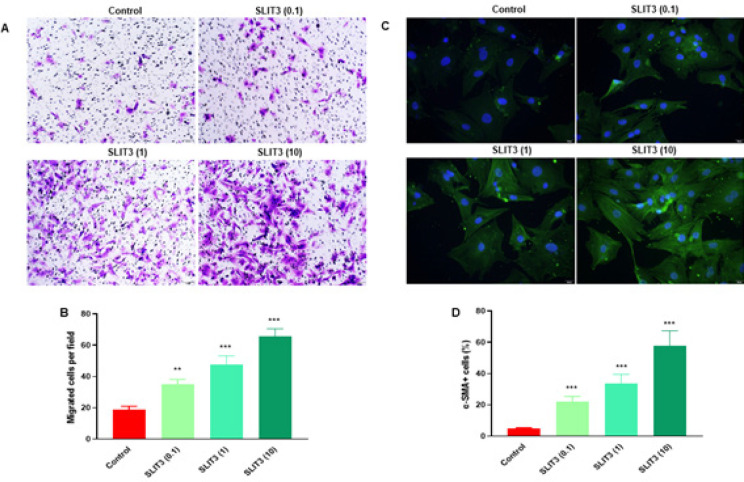
SLIT3 increased migration and differentiation of rat cardiac fibroblasts

A Transwell assay was performed to explore the migration ability of cardiac fibroblasts. Cardiac fibroblasts were treated with recombinant SLIT3 protein at 0.1, 1, and 10 μg/ml for 48 hr. Representative images showed that compared with the control group, SLIT3 increased the stained cells at 0.1, 1, and 10 μg/ml (Figure 3A). Quantitative analysis showed that SLIT3 significantly increased migrated cells in cardiac fibroblasts in a concentration-dependent manner (Figure 3B). To evaluate myofibroblast differentiation, immunofluorescence staining was used to detect α-smooth muscle actin (α-SMA) expression. Cardiac fibroblasts were treated with recombinant SLIT3 protein at 0.1, 1, and 10 μg/ml for 48 hr. SLIT3 treatment significantly increased α-SMA expression (Figure 3C). Quantitative analysis also revealed this effect in cardiac fibroblasts in a concentration-dependent manner (Figure 3D).

***SLIT3 suppression attenuated the Ang II-induced migration and collagen gene expression in cardiac fibroblasts***

**Figure 4:**
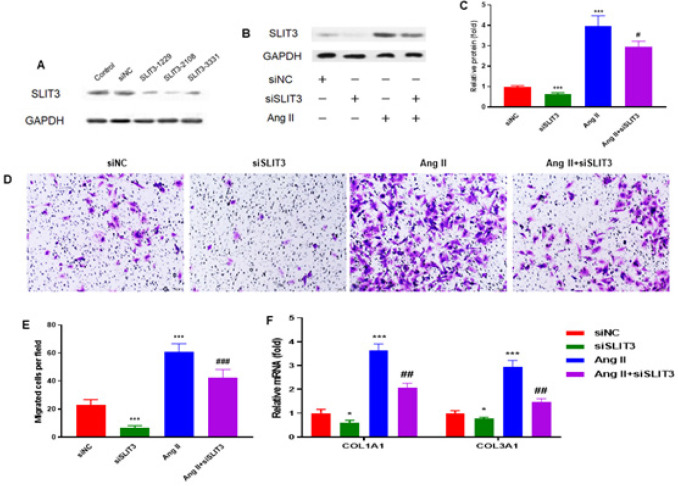
SLIT3 inhibition attenuated the Ang II-induced migration and collagen gene expression in cardiac fibroblasts

SLIT3 siRNA (siSLIT3) and control siRNA (siNC) were transfected to cardiac fibroblasts for 48 hr. Western blot was performed to assess protein levels of SLIT3; the 2108 sequence shows the best inhibitory effect (Figure 4A). Cardiac fibroblasts were transfected with SLIT3 or control siRNA, followed by incubation with 1 μM Ang II for 48 hr. SLIT3 inhibition markedly decreased SLIT3 protein expression in cardiac fibroblasts with or without Ang II incubation (n=3 in each group)(Figures 4B and 4C). A Transwell assay was performed to explore the migration ability of cardiac fibroblasts. Cardiac fibroblasts were treated with siSLIT3, Ang ll, and Ang ll+siSLIT3 for 48 hr. Representative images demonstrated that SLIT3 suppression significantly reduced SLIT3 protein expression in cardiac fibroblasts with or without Ang II incubation (n=3 in each group) (Figure 4D). This effect was also observed by quantitative analysis (Figure 4E). In addition, SLIT3 inhibition attenuated the Ang II-induced increase in mRNA expression of collagen I (COL1A1) and collagen III (COL3A1) in cardiac fibroblasts (n=3 in each group)(Figure 4F).

***SLIT3 suppression attenuated the Ang II-induced differentiation and ECM-related gene expressions in cardiac fibroblasts***

**Figure 5:**
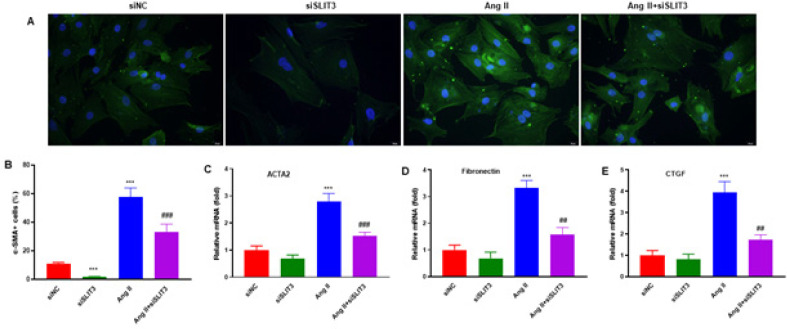
SLIT3 inhibition attenuated the Ang II-induced differentiation and ECM-related genes in cardiac fibroblasts

To investigate the effect of the Ang II-induced differentiation and ECM-related gene expressions in cardiac fibroblasts, cells were treated with siSLIT3, Ang ll, and Ang ll+siSLIT3 for 48 hr. SLIT3 inhibition significantly reduced α-SMA expression in cardiac fibroblasts with or without Ang II incubation (n=3 in each group)(Figure 5A). The same effect was also observed by quantitative analysis (Figure 5B). RT-qPCR was used to measure the mRNA expressions of ECM-related genes. SLIT3 inhibition attenuated the Ang II-induced increase in mRNA expression of ACTA2 (α-SMA) (Figure 5C), Fibronectin (Figure 5D), and CTGF (Figure 5E)(n=3 per group).

***SLIT3 suppression inhibited intracellular ROS generation and oxidative stress in Ang II-induced cardiac fibroblasts***

**Figure 6:**
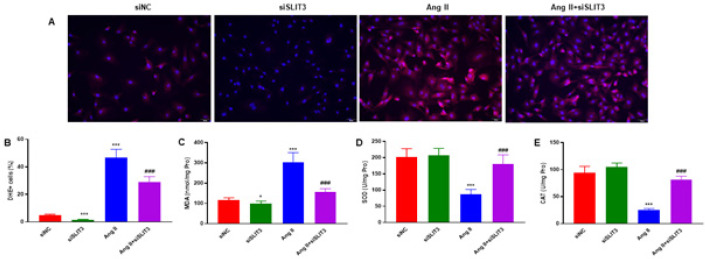
SLIT3 inhibition prevents intracellular ROS generation and oxidative stress in Ang II-induced cardiac fibroblasts

To investigate the inhibitory effect of SLIT3 on ROS production and oxidative stress, we carried out DHE staining to measure ROS levels in cardiac fibroblasts after Ang II administration. SLIT3 inhibition significantly reduced DHE expression in cardiac fibroblasts with or without Ang II incubation (Figure 6A). The same trends were observed by quantitative analysis (Figure 6B). Moreover, SLIT3 inhibition showed a more significant decrease in malondialdehyde (MDA) content than the LPS group (Figure 6C). The superoxide dismutase (SOD) and catalase (CAT) levels were significantly increased compared to the Ang II group (Figure 6D, 6E).

***SLIT3 activated RhoA/ROCK1 pathway in rat cardiac atrial fibroblasts***

**Figure 7:**
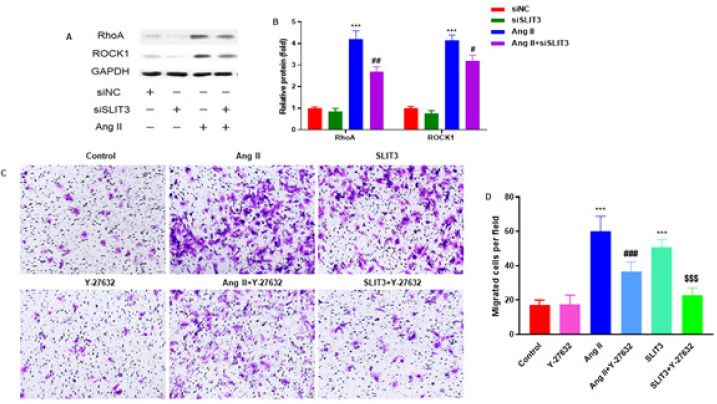
SLIT3 activated RhoA/ROCK1 pathway in rat cardiac atrial fibroblasts

Western blot was carried out to analyze the protein expression of RhoA and ROCK1 in cardiac fibroblasts of rats (Figure 7A). SLIT3 inhibition markedly decreased RhoA and ROCK1 protein expression in cardiac fibroblasts with Ang II incubation (n=3 in each group)(Figure 7B). The cardiac fibroblasts were pretreated with a ROCK inhibitor Y-27632 (10 μM) for 2 hr and then pretreated with Ang II (1 µM) and recombinant SLIT3 protein (1 μg/ml) for a further 48 hr. A Transwell assay was carried out to investigate the migration ability of cardiac fibroblasts (Figure 7C). ROCK inhibitor Y-27632 markedly attenuated the migration of cardiac fibroblasts stimulated by Ang II and SLIT3 (n=3 per group)(Figure 7D).

## Discussion

This study demonstrates that SLIT3 expression was enhanced in both Ang II-induced animal models and cardiac fibroblasts. SLIT3 had elevated migration and differentiation of rat cardiac fibroblasts. SLIT3 knockdown attenuated the Ang II-induced migration and collagen gene expression in cardiac fibroblasts. Ang II-induced differentiation and ECM-related gene expressions were inhibited by SLIT3 suppression. SLIT3 inhibition reduced intracellular ROS generation and oxidative stress in Ang II-induced cardiac fibroblasts. In addition, SLIT3 activated the RhoA/ROCK1 pathway in rat cardiac atrial fibroblasts. Therefore, SLIT3 could significantly affect cardiac fibrosis and fibroblast differentiation through the RhoA/ROCK1 pathway.

SLIT3 may have an impact on cardiac fibrosis and fibroblast differentiation. A recent study reported that SLIT3 deficiency reduced pressure overload-induced cardiac fibrosis and remodeling in mice (31). In addition, SLIT3 could regulate cardiac fibrosis by controlling the TGF-β/Smad signaling pathway and the fibrotic gene expressions (31). However, in our study, mice were infused with Ang II (2.0 mg/kg/day) or the same volume of saline for 28 days. Ang II significantly elevated the atrial fibrotic area (Figure 1B). The results also showed that SLIT3 and RhoA mRNA levels were significantly increased in Ang II-induced mice (Figure 1C, 1D, and 1E). In addition, SLIT3 protein levels were potentially elevated in Ang II-induced cardiac fibroblasts (n=3 in each group)(Figure 2C, 2D). Our result is consistent with the early report (26, 31), which showed that SLIT3 is highly expressed in cardiac fibroblasts.

To explore the migration ability of cardiac fibroblasts, cardiac fibroblasts were treated with recombinant SLIT3 protein and incubated for 48 hr. The results showed that SLIT3 increased the stained cells compared with the control group (Figure 3A) and significantly increased migrated cells in cardiac fibroblasts concentration-dependently (Figure 3B). Immunofluorescence staining was employed to identify the expression of α-SMA to assess myofibroblast differentiation. Recombinant SLIT3 protein was treated in cardiac fibroblasts for 48 hr. The expression of α-SMA was markedly elevated by SLIT3 treatment (Figures 3C and 3D).

Activated myofibroblasts are thought to be the main source of extracellular matrix (ECM) components that accumulate during cardiac fibrosis (32). Differentiation of cardiac fibroblasts to myofibroblasts is characterized by the expression of α-SMA and synthesis of ECM components, including collagen types I and III (33). *In vitro* and *in vivo* studies demonstrated that Ang II could increase collagen production and cardiac myofibroblast development (34, 35). However, in the present study, cardiac fibroblasts were treated with siSLIT3, Ang ll, and Ang ll+siSLIT3 for 48 hr. The result showed that in cardiac fibroblasts with or without Ang II treatment, SLIT3 suppression dramatically decreased SLIT3 protein expression (Figures 4D and 4E). In addition, SLIT3 suppression reduced the Ang II-induced increase in collagen I (COL1A1) and collagen III (COL3A1) mRNA expression in cardiac fibroblasts (Figure 4F). Moreover, the Ang II-induced improvement in the mRNA expression of ACTA2 (α-SMA), Fibronectin, and CTGF was reduced by SLIT3 suppression (Figures 5C, 5D, and 5E).

There is accumulating research support that abnormal cardiac remodeling is significantly influenced by oxidative stress (36). In response to TGF-β1, ROS are required to produce the α-SMA protein and the differentiation of myofibroblasts (37). In our investigation, we found that Ang II markedly boosted the production of intracellular ROS. This observation is consistent with other reports (38, 39). However, our present study results showed that inhibition of SLIT3 dramatically decreased the expression of DHE in cardiac fibroblasts (Figures 6A and 6B). In addition, malondialdehyde (MDA) levels decreased more noticeably with SLIT3 inhibition (Figure 6C). In comparison to the Ang II group, the levels of catalase (CAT) and superoxide dismutase (SOD) were considerably higher (Figure 6D, 6E). The impact of SLIT3 on Ang II-stimulated ROS production in cardiac fibroblasts remained unknown before our current investigation.

The expression of RhoA and ROCK in several myocardial fibrosis models has been shown in earlier investigations. The RhoA/ROCK pathway influences critical mediators of several pro-fibrotic processes (40-42). However, our research consistently demonstrated that SLIT3 decreased the expression of α-SMA, collagen I, collagen III, RhoA, and ROCK1 in cardiac fibroblast tissues induced by Ang II. In addition, *in vitro* tests further show that SLIT3 can suppress the production of α-SMA, collagen I, collagen III, RhoA, and ROCK1 and prevent the Ang II-induced conversion of fibroblasts into myofibroblasts. Therefore, the RhoA/ROCK1 signaling pathway may be inhibited by SLIT3 to ameliorate Ang II-induced fibrosis.

There are several limitations in the present study. (a) In clinical research, the mechanisms of SLIT3 are complicated and ambiguous. However, the main goals of this study were to assess how SLIT3 promotes cardiac fibrosis and fibroblast differentiation in mice. (b) Even though SLIT3 could stimulate cardiac fibrosis and fibroblast differentiation, regulating them might not be possible if they already exist. However, SLIT3 might be a critical preventative and therapeutic approach for cardiac fibrosis and fibroblast differentiation in pre-clinical and clinical settings, although further investigation is required to validate this. 

**Table 1:** List of primer sequences used for the current research

**Genes**	**Forward primer (** **5′-3′)**	**Reverse primer (** **5′-3′)**
Mouse SLIT3	AGTTGTCTGCCTTCCGACAG	TTTCCATGGAGGGTCAGCAC
Mouse RhoA	GAGTTGGACTAGGCAAGAAACTC	ACCCAAACCCTCACTGTCTTC
Rat SLIT3	GGCAAGGACTCCTATGTGGA	GGGTCGTTGTCTCCCTTGTA
Rat Collagen I	GCCTCAGCCACCTCAAGAGA	GGCTGCGGATGTTCTCAATC
Rat Collagen III	CCAGGACAAAGAGGGGAACC	CCATTTCACCTTTCCCACCA
Rat ACTA2 (α-SMA)	CTATTCCTTCGTGACTACT	ATGCTGTTATAGGTGGTT
Rat Fibronectin	GGATCCCCTCCCAGAGAAGT	GGGTGTGGAAGGGTAACCAG
Rat CTGF	AGAGTGGAGATGCCAGGAGA	CACACACCCAGCTCTTGCTA
Rat GAPDH	CCCCCAATGTATCCGTTGTG	TAGCCCAGGATGCCCTTTAGT

Mice were infused with Ang II (2.0 mg/kg/day) or the same volume saline for 28 days. (A) Masson trichrome staining and immunocytochemistry of SLIT3 in the right ventricular myocardium (200×). (B) Cardiac fibrosis was evaluated by calculating the percentage of fibrotic area. (C) SLIT3 and RhoA mRNA levels were significantly elevated in ventricular muscle from mice with Ang II infusion compared with that from control mice (n=4 in each group). (D) Representative blots of SLIT3 and RhoA protein by western blot analysis. (E) SLIT3 and RhoA protein levels were significantly increased in ventricular muscle from mice with Ang II infusion compared with that from control mice (n=4 in each group)

(A) Cardiac fibroblasts were isolated from 1 to 2-day newborn SD rats (lactating mice born within three days) using differential adhesion method and were identified using Vimentin staining (400×). (B) The cardiac fibroblasts were treated with Ang II at 0.1, 1, and 10 μM for 48 hr. SLIT3 mRNA levels were significantly elevated in cardiac fibroblasts after Ang II treatment in a concentration-dependent manner. (C) Representative blots of SLIT3 protein by western blot analysis. (D) SLIT3 protein levels were significantly increased in Ang II-induced cardiac fibroblasts (n=3 in each group). Data are shown as mean±SD and analyzed by ANOVA. *P<0.05, **P<0.01, ***P<0.001 vs the control group

Cardiac fibroblasts were treated with recombinant SLIT3 protein at 0.1, 1, and 10 μg/ml for 48 hr. (A) Cell migration was measured by Transwell migration assay (200×). (B) Quantitative analysis of the number of migrated cells per field (n=3 in each group). (C) Representative images of immunofluorescence for α-SMA (400×). (D) Quantitative analysis of the percentage of α-SMA positive cells (n=3 in each group). Data are shown as mean±SD and analyzed by ANOVA. **P<0.01, ***P<0.001 vs the control group

(A) Western blot was performed to assess protein levels of SLIT3, and the 2108 sequence shows the best inhibitory effect. (B) Representative blots of SLIT3 protein by western blot analysis. (C) SLIT3 inhibition markedly decreased SLIT3 protein expression in cardiac fibroblasts with or without Ang II incubation (n=3 in each group). (D) Representative images of migrated cells by Transwell. (E) Quantitative analysis of the number of migrated cells per field (n=6 in each group). (F) SLIT3 inhibition attenuated the Ang II-induced increase in mRNA expression of collagen I (COL1A1) and collagen III (COL3A1) in cardiac fibroblasts (n=3 in each group). Data are shown as mean±SD and analyzed by ANOVA. *P<0.05, ***P<0.001 vs control group; ^##^*P<*0.01, ^###^*P<*0.001 vs Ang II group

(A) Representative images of immunofluorescence for α-SMA (400×). (B) Quantitative analysis of the percentage of α-SMA positive cells (n=3 in each group). RT-qPCR was used to measure the mRNA expressions of ECM-related genes. SLIT3 inhibition attenuated the Ang II-induced increase in mRNA expression of (C) ACTA2 (α-SMA), (D) Fibronectin, and (E) CTGF (n=3 per group). Data are shown as mean±SD and analyzed by ANOVA. ***P<0.001 vs control group; ^##^*P<*0.01, ^###^*P<*0.001 vs Ang II group

(A) Representative image of intracellular ROS detected by DHE (200×). (B) Quantitative analysis of DHE-positive cells among all cardiac fibroblasts (DAPI staining). The oxidative stress marker was detected in cell lysate for (C) MDA, (D) SOD, and (E) CAT (n=3 per group). Data are shown as mean±SD and analyzed by ANOVA. *P<0.05, ***P<0.001 vs control group; ^###^*P<*0.001 vs Ang II group

(A) Representative western blot bands of RhoA and ROCK1 protein in rat cardiac fibroblasts. (B) SLIT3 inhibition markedly decreased RhoA and ROCK1 protein expression in cardiac fibroblasts with Ang II incubation (n=3 in each group). The fibroblasts were pretreated with a ROCK inhibitor Y-27632 (10 μM) for 2 hr and then pretreated with Ang II (1 µM) and recombinant SLIT3 protein (1 μg/ml) for a further 48 hr. (C) Representative images of migrated cells by Transwell. (D) ROCK inhibitor Y-27632 markedly attenuated migration of cardiac fibroblasts stimulated by Ang II and SLIT3 (n=3 per group). Data are shown as mean±SD and analyzed by ANOVA. ***P<0.001 vs control group; ^###^*P<*0.001 vs Ang II group; ^$$$^*P<*0.001 vs SLIT3 group

## Conclusion

Our accumulated data suggest that SLIT3 significantly regulates Ang II-induced cardiac fibrosis and fibroblast differentiation via the RhoA/ROCK1 signaling pathway. Further study is needed to verify the effectiveness of SLIT3 as a preventative and therapeutic approach for cardiac fibrosis and fibroblast differentiation in pre-clinical and clinical settings.

## Authors’ Contributions

All authors significantly contributed to this study. X Z and B T performed experiments and wrote the manuscript; X C performed experiments, revised the manuscript, and provided statistical analysis; Z N conceived the idea and designed and supervised the study.

## Conflicts of Interest

The authors declare no conflicts of interest, financial or with other people or organizations.
